# Development of a 3D Printed Double-Acting Linear Pneumatic Actuator for the Tendon Gripping

**DOI:** 10.3390/polym13152528

**Published:** 2021-07-30

**Authors:** Ivan Grgić, Vjekoslav Wertheimer, Mirko Karakašić, Željko Ivandić

**Affiliations:** 1 Mechanical Engineering Faculty in Slavonski Brod, University of Slavonski Brod, Trg Ivane Brlić Mažuranić 2, 35000 Slavonski Brod, Croatia; mkarakasic@unisb.hr (M.K.); zivandic@unisb.hr (Ž.I.); 2 Faculty of Medicine Osijek, Josip Juraj Strossmayer University of Osijek, Joispa Hutlera 4, 31000 Osijek, Croatia; vjekoslav.wertheimer@gmail.com; 3 Department of Orthopedics and Traumatology, Osijek University Hospital, 31000 Osijek, Croatia

**Keywords:** linear pneumatic actuator, 3D printing, fused deposition modeling, tendon gripping

## Abstract

The lack of standardization in tissue testing procedures results in a variety of custom-made devices. In the case of the determination of the mechanical properties of tendons, it is sometimes necessary to adapt the existing laboratory equipment for conducting experiments when specific commercial equipment is not applicable to solve issues such as proper gripping to prevent tendon slipping and rupturing, gripping control and manoeuvrability in case of tendon submerging and without contamination of the testing liquid. This paper presents the systematic development, design, and fabrication using 3D printing technology and the application of the double-acting linear pneumatic actuator to overcome such issues. It is designed to do its work submerged in the Ringers’ solution while gripping the tendon along with the clamps. The pneumatic foot valve unit of the Shimadzu AGS-X tensile testing machine controls the actuator thus preventing Ringers’ solution to be contaminated by the machine operator during specimen set-up. The actuator has a length of 60 mm, a bore of 50 mm, and a stroke length of 20 mm. It is designed to operate with an inlet pressure of up to 0.8 MPa. It comprises the cylinder body with the integrated thread, the piston, the piston head, and the gripper jaw. Fused deposition modeling (FDM) has been used as the 3D printing technique, along with polylactic acid (PLA) as the material for 3D printing. The 3D printed double-acting linear pneumatic actuator was developed into an operating prototype. This study could open new frontiers in the field of tissue testing and the development of similar specialized devices for medical purposes.

## 1. Introduction

Product design and development sometimes touch the boundaries of their usual field of activity, occasionally jumping into other scientific disciplines such as medicine to provide answers. Involving designers in solving tasks in which medicine is no longer competitive requires an additional effort that includes getting knowledge from fields the designer is not familiar with. Involving medical experts at certain stages of the design process also becomes a necessity. Such collaboration results in products that contribute to an understanding of human health. Most of the knowledge about the biomechanical properties of tendons and ligaments comes from the tensile testing procedures in which the specimen is placed in the jaws of the machine and tensile force is applied to the breaking point [1,2,3,4,5,6]. Because of soft tissue viscoelastic characteristics and low friction between the clamp material and wet soft tissues, it was hard to hold them properly at in vitro loads and loading speed. Excessive compression on the soft tissue will elevate stress around the contact area, which leads to rupture before target loads are achieved. Less compression will result in slippage [7]. Furthermore, during conducting experiments, it is necessary to prevent tissues from dehydration by constantly spraying or submerging them to get valid results. Researchers have attempted to deal with such problems in many ways. Hangody et al. [8] evaluated five different types of fixation devices: surgical thread (Premicron 3), wire mesh, clamp, cement fixation, Shi’s clamp and Shi’s modified frozen clamp for an Instron loading machine. In this paper, attempts were presented to find a suitable fixation technique for allograft endurance testing and adapt it to be compatible with the Instron loading machine. Shi’s modified frozen clamp was the only device that resulted in proper, stable fixation during endurance testing. This fixation device was presented in [9]. It has lateral block boards and asymmetric tooth jaws. The titanium alloy lateral block boards were used to prevent the soft tissues from being pushed out during compression, while the nylon asymmetric tooth jaws were used to grip and hold the soft tissues. The ability of this new type of clamp was tested by stretching five bovine tendons to failure on the tensile and compression testing machine; none of them showed slippage before failure, and the maximum tensile force was 6.87 kN. This non-frozen asymmetric jaw clamp was developed for gripping tendons in the dynamic foot and ankle simulation tests, but can also be used for other in vitro tests, such as dynamic hip and knee tests. Cheung and Zhang [10] introduced a specially made serrated jaw clamp for tendon gripping. They pointed out that the non-frozen tendon clamps require only manual tightening with the screws, without the need for a complicated freezing system and procedure. An expensive and massive device is not needed to grip the tendon and to avoid slippage of the tendon due to the thawing of the tissue during the experiment. Other advantages of this clamp are its relatively small size and weight, low labour requirement and short fabrication time due to the commercially available serrated plastic material. The other clamping techniques can be found in [11,12,13,14].

The tendon gripping device has been reported as a patent by McCallion [15]. The tendon gripping device includes a first opening and a second opening to facilitate the passage of a tendon through the device. The jaw bundle includes a plurality of elongated members that enclose the tendon, are generally wedge-shaped and are complementary to an inner conical wall of the housing.

Ng et al. [16] have used various gripping methods including serrated jaws, sandpaper, frozen ends and air-dried ends. They have found that the cardboard-lined pneumatic grippers with 1 kN (Shimadzu Europa GmbH, Albert-Hahn-Strasse 6-10, 47269 Duisburg, F.R. Germany) provided adequate grip without noticeable slippage and damage to the tendons. When the cardboard-lined pneumatic grips were used, the stress concentration at the interface between the grips and the specimen was significantly reduced.

From the previously reported studies, it is clear that standard testing equipment still needs improvement and the development of new products for medical use continues. It can be seen that the tendon wetting, slipping and rupturing can be successfully solved by choosing the appropriate combination of materials and gripping techniques; however, there is no clear statement about what kind of equipment should be used as a standard for the tensile testing of the tendons. This opens up the search for other materials and techniques which could possibly be used for tensile testing of the tendons and have the potential to be standardized in the future. Therefore, to satisfy the requests of the wetted tendon during tests by keeping it within jaws without slippage and tissue rupture in the clamping area, our focus fell to 3D printing. With the materials used for the 3D printing, the corrosion appearance can be avoided, so while wetting the tissue is no longer a problem, material porosity should be considered. The gripping technique is a major challenge because it depends on many parameters, starting from the component design, component 3D printing parameters, the direction of a 3D printing, the strength of 3D printed parts, etc. The slippage has the same challenges. Furthermore, to conduct the experiment by keeping the tendon submerged in the liquid to provide adequate wetting, but to reduce the human effort in the gripping procedure to prevent the contamination of the testing liquid request a deep analysis and it is not yet properly investigated. The necessity for this study came from the collaboration with the medical experts (in the arthroscopy and orthopaedic fields). Particularly, the lack of specific laboratory equipment for human tendon tensile testing led to this study. It was necessary to develop a modular technical system that will be integrated into the existing laboratory equipment (Shimadzu AGS-X 10 kN tensile test machine) to conduct the tensile tests of human gracilis and quadriceps tendons. We chose the FDM technology to conduct this study by 3D printing the actuator and its components.

No work has been found presenting the use of the 3D printed pneumatic actuator involved in gripping/clamping the tendons.

3D printing technology has been widely used in the industry, and serve in many practical applications. Ning et al. [17] reported that FDM is one of the most popular additive manufacturing techniques. According to them, it offers advantages such as low cost, minimal wastage and ease of material change, and it is used for fabricating thermoplastic parts that are mainly used as rapid prototypes for functional testing. According to Reddy et al. [18], FDM is mostly used to develop function prototypes and in few applications, it is useful to manufacture end-use products. Since the presented actuator is to be developed up to the level of functional prototype, the FDM technology was chosen for the manufacturing process.

Krause and Bhounsule [19] presented a linear pneumatic actuator fabricated using 3D printing technology with electronic sensors for force and position control. PolyLactic Acid (PLA) material was used for 3D printing. The thickness of the cylinder body was chosen to be 1.143 mm. The maximum printing resolution was 0.0125 mm. A thin layer of material was removed in a mechanical post-processing procedure to produce a smooth surface. The authors in [20] presented the design and fabrication of a 3D-printed double-acting miniature linear pneumatic actuator, type On-Off. The actuator has an overall length of 80 mm, a bore size of 15 mm and a stroke length of 20 mm. The total weight is 0.012 kg and it produces a peak output power of 2 W at an input air pressure of 0.27 MPa. The utility of the actuator is demonstrated in a series of jumping experiments with the actuator and by incorporating the actuator into a bouncing robot inspired by the Disney/Pixar Luxo lamp. They have concluded that 3D printed pneumatic actuators combine the high performance of pneumatics with the lightweight of plastics and the structural strength provided by the selective placement of metal parts, offering a promising actuator for robotic applications. However, there are a large number of works presenting 3D printed pneumatic actuators in soft robotics that will not be specifically mentioned here.

Although 3D printing has been used to manufacture soft pneumatic actuators that operate at relatively low supply pressures, 3D printing has not been used to manufacture conventional pneumatic actuators, such as the piston-cylinder assembly, that can operate at standard pneumatic supply pressures. The fabrication of 3D printed actuators using plastic filaments and hobbyist printers poses a significant challenge, as the low resolution of hobbyist printers leads to the following problems: parts with limited strength, rough surfaces (high friction) that reduce efficiency, a considerable backlash that leads to leakage and anisotropy of parts that leads to directional strength [19].

In this paper, we present our experience in the development of the pneumatic actuator, starting with the selection of the 3D printing parameters, followed by the design and the pressure testing of the samples. We also present a special tool for machining the inner cylinder body. This study is an extension of previously published work [21]. Finally, conclusions were drawn and the final design is proposed at the functional prototype level.

## 2. Materials and Methods

All 3D models were constructed and exported as STL (stereolithography) files and post-processed in the free software Ultimaker Cura [22]. Three-dimensional printing was performed using the hobbyist Creality (Shenzhen Creality 3D Technology Co., Ltd., Shenzen, China) Ender-3 Pro 3D printer.

**Figure 1:**
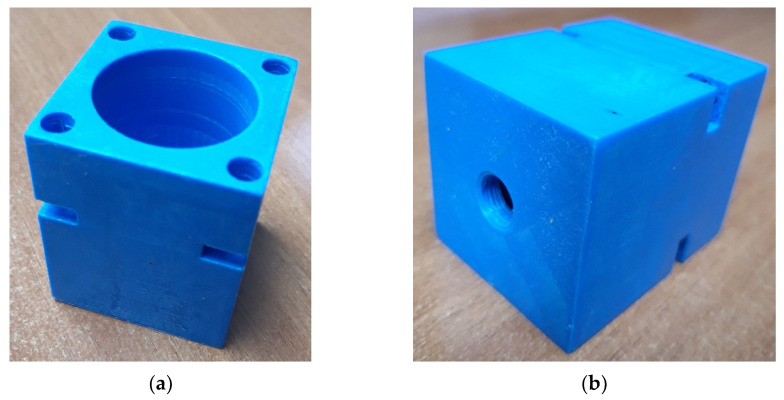
(**a**) Front side of the cylinder; (**b**) Backside of the cylinder with a hole with thread for the pneumatic hose connection.

In order to test the capabilities of this 3D printer and its printing quality, the printing of the test patterns in different designs was started. Each of the tested patterns was connected to the pneumatic tube via the hole with the integrated thread. An example of a tested pattern of a cylinder is shown in Figure 1.

**Figure 2:**
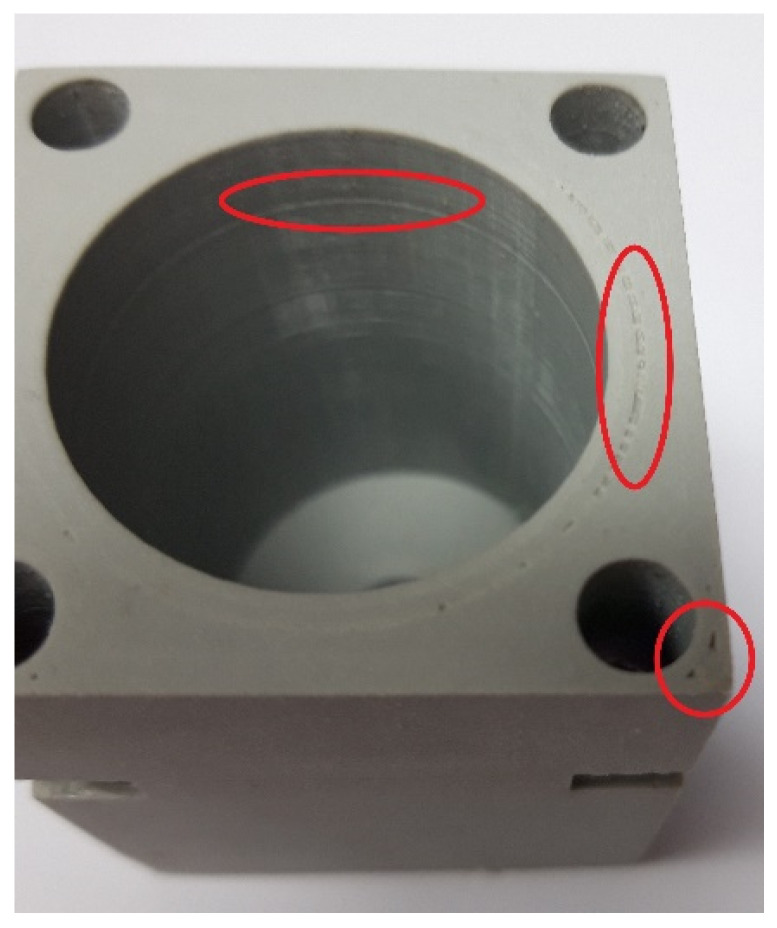
Errors on 3D printed test cylinder.

At the front of the cylinder, the holes for the insertion screws were made in the cap of the cylinder and tightened with a nut inserted into the notch on the body of the cylinder. At the rear, the threaded hole was made to connect the pneumatic hose to the flange so that compressed air could be supplied from the compressor. A rubber gasket was placed between the cylinder body and the cap to prevent leakage between them. It was started with the standard 3D printing parameters recommended by the 3D printer manufacturer. As expected, the model presented had numerous shortcomings. The biggest problem was the poor print quality of the 3D printer. Figure 2 shows the gaps between the layers caused by the leakage of compressed air from the cylinder. The defects are visible between the layers on the inside of the cylinder and the top surface. Subsequently, the 3D printing parameters were varied to avoid similar defects and to achieve the highest quality of the inner surface of the cylinder for a uniform piston stroke. Every 3D printer has its advantages and disadvantages, and they can affect the quality of the 3D printed model since it was necessary to establish the parameters for 3D printing [23,24]. The varied parameters are the layer height, shell thickness, infill percentage, infill overlap percentage between the wall and shell during printing, melting temperature of the material during printing and printing speed. Explanations of each parameter can be found below.

- Layer height

According to the technical information provided by the manufacturer of the 3D printer used, the specified print quality is in the range of 0.1–0.4 mm. Through the numerous experiments of 3D printing, it was found and assumed that the layer height of 0.01 mm is chosen for the 3D printing quality. It can be seen that the limits of the declared 3D printing quality have been exceeded.

- Shell Thickness

**Figure 3:**
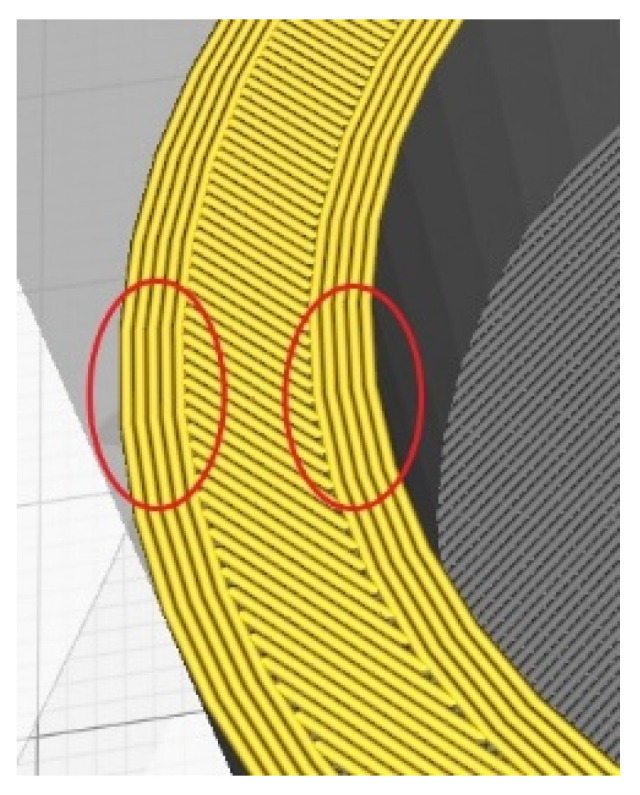
Outer and the inner thickness (red markers) with the cylinder body in between.

Shell thickness refers to the thickness of the outer and inner layers on the model, followed by the filling of the body of a model. It depends on the diameter of the nozzle, which is in this case 0.4 mm, meaning that if the shell thickness is, say, 2 mm, the 3D printer will make five passes (five concentric circles). In this case, the shell thickness of the outer and inner sides of the cylinder was 1 mm, and in between is the 6 mm of the cylinder body (Figure 3). The actuator thickness is 7 mm overall.

- Overlap Percentage

To avoid the occurrence of gaps between the boundary layers separating the shell and the cylinder body area, the Cura software provides the option of overlapping by specifying the percentage of overlap. This means that when 3D printing the body area of the cylinder, the nozzle stroke will overlap into the shell area to the extent of the defined percentage. In this case, it was set to 40%. A body infill density of less than 100% was not considered.

- Printing Temperature

The recommended printing temperature of PLA material is 185–215 °C. At lower temperatures, weaker interlayer occupancy has been observed due to insufficient sinking and bonding of the layers per pass. At higher temperatures there is better bonding, but also a “dirty” print, where the material melts and expands more and causes greater deformability, disrupting the standard shape and fouling of the nozzle. The material burns and contamination occurs between the layers. In this case, the selected temperature for 3D printing the actuator components was 200 °C with a heating bed temperature of 70 °C.

- 3D Printing Speed

High printing speeds make it impossible for the molten material to settle in the intended location in time, although the extrusion speed can be increased and the error corrected, which requires a very sophisticated parametric analysis. Based on the experience, the selected infill speed was set at 20 mm/s with a wall printing speed of 10 mm/s. Table 1 represents the summary of the 3D printing parameters.

### 2.1. Samples Test and Mechanical Post-Processing

**Figure 4:**
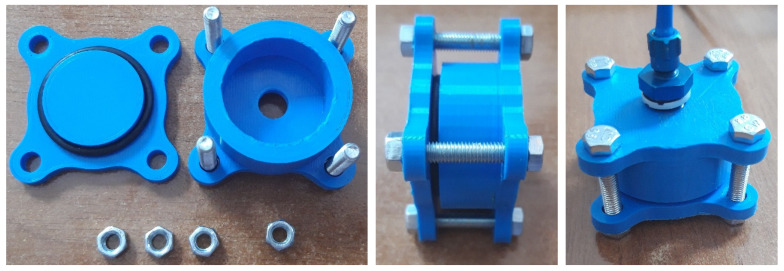
The preparation of the assembly to be connected to a pneumatic hose and tested submerged in the water container.

In correlation to the preliminary design, the geometry was changed and a new sample of a cylinder was designed. 3D printing was performed with the specified parameters and a very smooth and clean surface was obtained. The density between the layers was very satisfactory. The bolts were used together with a gasket to seal the cylinder with its head and the pneumatic tube also on the backside. The assembly of a tested sample is shown in Figure 4. The pneumatic hose was connected to a test cylinder and immersed in a water tank along with it. We gradually increased the pressure from 0 to 0.5 MPa, and problems with sealing were encountered.

**Figure 5:**
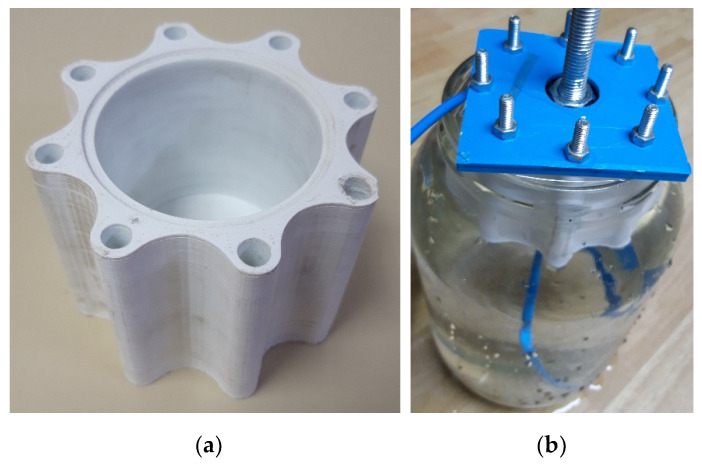
(**a**) The new cylinder body; (**b**) The submerged assembly in the water container—no leaking was found.

During the analysis, it was found that the bolt tension caused a small deformation of the cylinder head and the gasket distribution was not well distributed, causing air leakage. Reducing the bolt tension was not an option; therefore, the number of bolts was increased, and a new cylinder body was fabricated (Figure 5a) with an inner diameter of 50 mm. A custom-made cylinder cap was designed and connected with the eight M5 × 80 mm bolts with the gasket for sealing insurance between the cylinder body and the cap. The TDOP 50 [25] piston head was used connected to the M10 × 100 mm bolt, which served as a guiding piston. The same test setup was run several times and, finally, no leakage was detected (Figure 5b) and parameters were confirmed. The next step in the development process was to deal with the inner surface of the cylinder body for smooth movement of the piston head.

**Figure 6:**
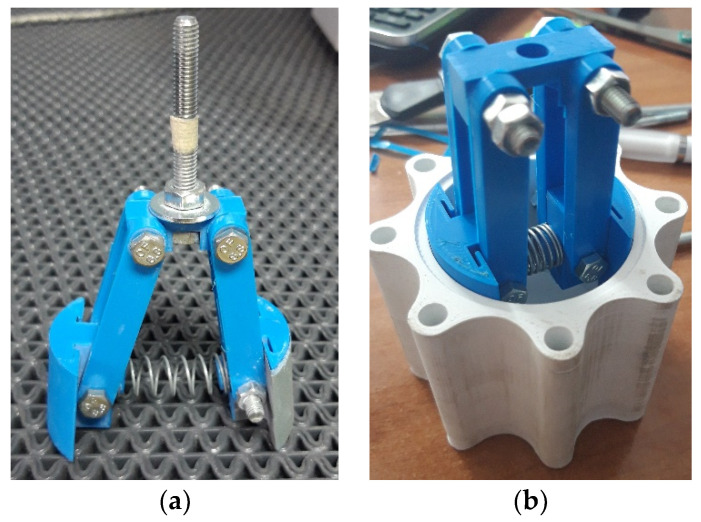
(**a**) The tool for the sanding process; (**b**) The preparation of the cylinder body and the tool for the sanding process.

In this step, the 3D printing process was also used to 3D print the parts of a specially designed tool for the sanding process, which is shown in Figure 6. It consists of spring-loaded arms connected in pairs with bolts. The rounded elements have a radius of 25 mm, which corresponds to the inner radius of the previously tested cylinder. Water sandpaper was wrapped around the rounded elements and the tool was inserted into the cordless drill, which was moved along the length of the cylinder on its inner side. We started with 80 grit (coarse) water sandpaper and finished with 2500 grit (fine) water sandpaper. Ample water was used during sanding to avoid deformation and damage to the cylinder [19]. After the sanding process, the cylinder was repressurized using the supplied 50 mm piston head TDOP 50 [25]. The sanding procedure has been done for the piston and the internal surface of the end cap of the cylinder as well. The movement of the piston occurs at 0.05 MPa. It was very smooth without steps and it was found that the inner surface of the cylinder was sufficiently polished.

### 2.2. Preliminary Test

**Figure 7:**
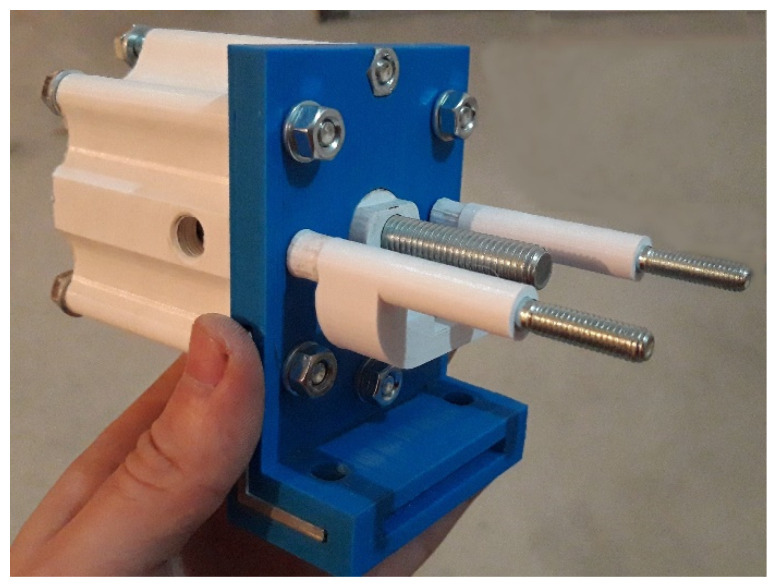
Preliminary design of the 3D printed actuator.

**Figure 8:**
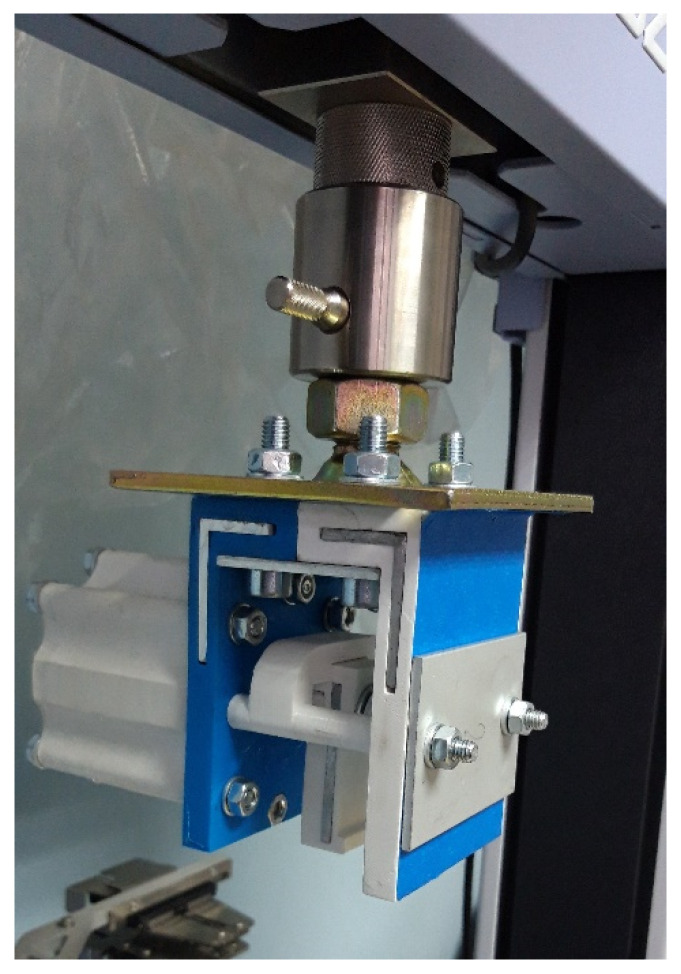
Fully assembled pneumatic cylinder with the jaws and mounted on the tensile testing machine.

After establishing the 3D printing parameters and a preliminary design of a cylinder shape and a size, as well as the sanding process for mechanical finishing, the next step was to look at the workspace of the Shimadzu AGS-X machine to see what the possibilities were, what the requirements were and what impact they might have on the existing cylinder design. The upper and lower machine gripps were reassembled and measured. The mounting points were analyzed and so was the pneumatic system of the machine. Finally, the preliminary design of the new double-acting linear pneumatic actuator was presented and fully assembled and mounted on the Shimadzu AGS-X machine, shown in Figure 7 and Figure 8.

The experiments conducted by using this presented preliminary design of a 3D printed pneumatic cylinder with porcine tendons can be seen in [26]. The results in the published study have been used for preliminary design validation. In addition, the same interpretation is valid for the validation in this study as well since the same actuator has been used for gripping the proposed clamps in [26].

The recorded key advantages and disadvantages were as follows

Advantages:

- Easy to install and managing;
- Durability—no failures;
- Light construction (PLA and aluminium parts);
- Easy assembling and reassembling;
- In case of a failure, fast replacement of parts;
- No need for ordering parts from the manufacturer;
- Can be immersed in liquid.

Disadvantages:

- Structural reinforcement with aluminium parts;
- Number of bolts connections;
- Design of surrounding parts—better visibility of the tendon clamps is needed for easier placing in the jaws;
- Partial air leaking occurs over time—a thin silicon layer is applied.

## 3. Final Actuator Design

The holes together with the bolts have been removed and the body of the cylinder is completely rounded. The body length of 60 mm, the bore dimension of 50 mm, the stroke length of 20 mm and the reinforced piston with the bolt have not been changed. For the connection of the cylinder with the end cap, the inner tube fine thread G 1¾ was modeled. The end cap design was changed from a square shape to a rounded shape. One side has an external pipe thread to connect the cylinder body and an internal housing for sealing and moving the piston. The L-section aluminium reinforcements were removed and replaced with the new base design. The jaws were left unchanged, as well as the jaw guiding part to prevent the jaws from rotating. A thin silicon layer was applied to the outer surface of the cylinder and its end cap.

**Figure 9:**
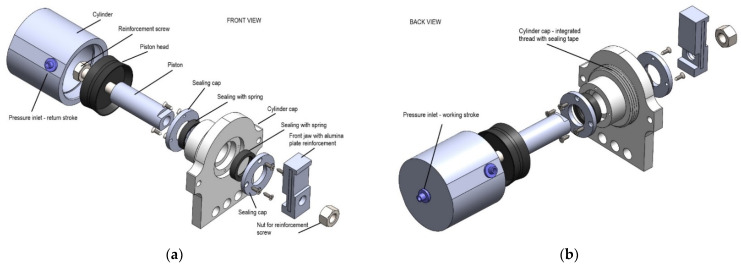
The actuator: (**a**) Front exploded view; (**b**) Back exploded view.

**Figure 10:**
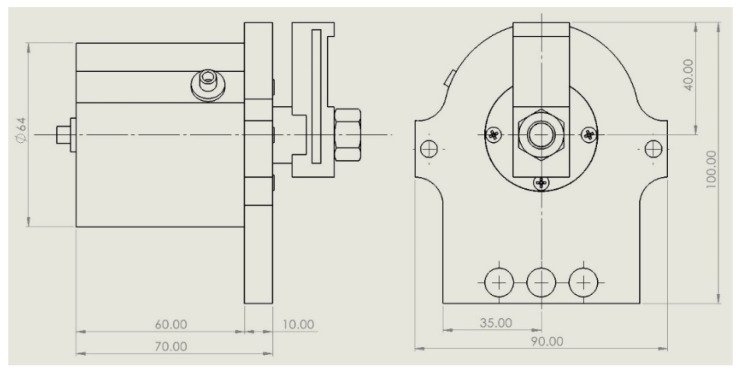
The main dimensions of the cylinder geometry.

In Figure 9, the proposed design of the 3D printed double-acting linear pneumatic actuator is given in detail. The main geometry dimensions are presented in Figure 10.

**Figure 11:**
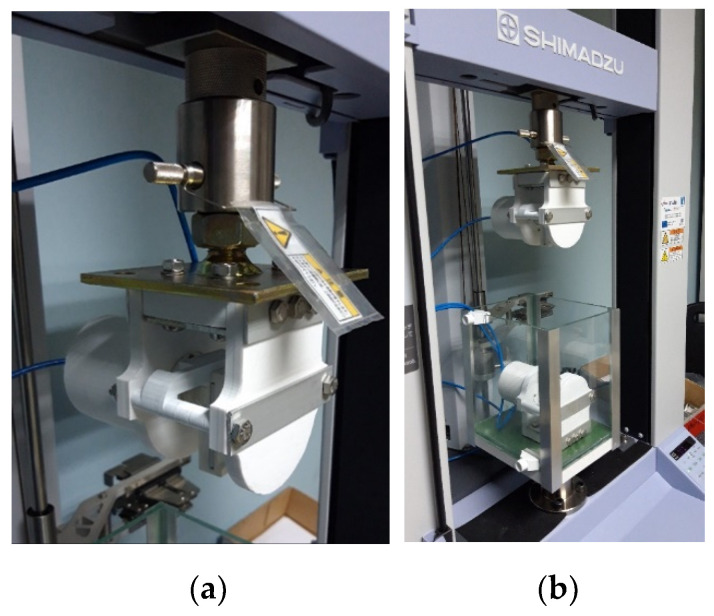
(**a**) The actuator mounted on the tensile test machine; (**b**) The actuators in their workspace.

The assembled and mounted actuator on the machine is presented below in Figure 11.

## 4. Results

To validate the design and functionality of the proposed actuator, we decided to perform the stress-relaxation test of tendons. The experiment setup and tendons preparation can be found in [21]. Moreover, the same interpretation applies to the validation of the actuator design in this study, as the same actuator was used for gripping the proposed clamps along with the human gracilis and quadriceps tendons.

**Figure 12:**
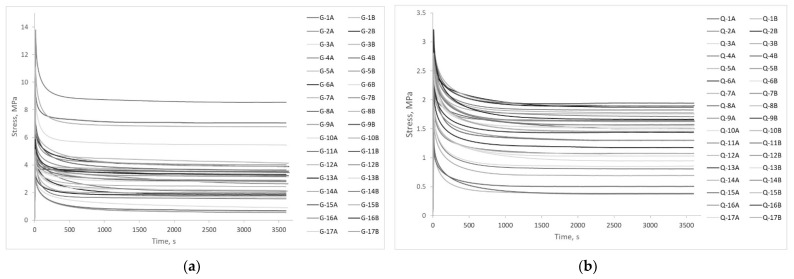
(**a**) Stress-relaxation test of gracilis tendons; (**b**) Stress-relaxation test of quadriceps tendons.

The stress-relaxation test is a long-lasting test of the specimens used to determine a time-dependent decrease in stress under a constant strain. The 34 human gracilis (denoted as G-1A to G-17B) and 34 quadriceps (denoted as Q-1A to Q-17B) tendons are subjected to the stress-relaxation test. Each sample was subjected to the stress relaxation test for 3600 s at 3% strain (Figure 12). The pressure in the actuator was set to 0.3 MPa. During this period, the pressure drop on the air compressor gage is monitored. However, if a pressure drop occurs, the stress-relaxation curve would not behave properly because the gripping will not be sufficient to withstand the constant load. Overall, 68 human tendons were tested and no pressure drop was found. Tendon gripping went without issues and stress-relaxation curves show no changes. Additional stress-relaxation data analysis will not be performed in this paper because it is a part of future work.

## 5. Discussion

The possibilities of implementing an innovative device fabricated using FDM technology to operate within conventional laboratory equipment to perform experiments on human tendons are investigated. Requirements such as wetting of tissue, suitable clamping technique, the inadequacy of laboratory equipment for tissue testing, expensive special tissue testing systems, commercially available actuators and their possibilities of immersion in the fluid, etc. motivated us to deal with this project. From our experience with hobby-grade 3D printers, we emphasize that it is necessary to conduct preliminary experiments to determine the 3D printing parameters, to see what capabilities such 3D printers have to meet and then to consider whether to proceed with the research or to look for other solutions. The experiments prove the previous statement and the 3D printing parameters can be determined without the need for other solutions.

Several attempts were made to determine the final design of the actuator. The trials show several problems we have to deal with, such as the low resolution of the 3D printer resulting in a rough surface. Therefore, it is proceeded with the post-processing procedure by sanding the inner surface of the cylinder body similar to what was reported in [19,20]. The special tool was designed and successfully used. The pores in the 3D printed part were the next problem. The problems with air leaks occurred during prolonged compressive loading through the cylinder wall. Immersing the 3D printed parts in acetone was not an option because the acetone does not dissolve the PLA [19]. It was necessary to apply a thin layer of silicone to the outer surface of the cylinder body and its end cap. In the future, acrylonitrile butadiene styrene (ABS) should be considered as a material for 3D printing because it seals the pores of the 3D printed model, preventing air from leaking through the cylinder wall [19]. Studies such as [27], which reported on modeling and 3D printing of porous structures, were considered to get an idea of modeling the actuator body structure that could help with leakage problems, but ultimately, this was not an option.

To be able to operate at high pressure, the actuator needs reinforcement parts. Metal inserts were also reported in [19].

To keep the tissue moist and prevent it from drying out, the actuator must be able to be operated immersed in the test fluid. For this reason, it was designed as a double-acting actuator. The working stroke and the return stroke are independent of each other and controlled outside the working area of the machine by pneumatic foot pedals. Contamination of the test fluid was kept to a minimum. The application tests of the actuator were carried out with the porcine [26] and human gracilis and quadriceps tendons [21]. It was necessary to select the appropriate cylinder diameter and to determine the gripping force (axial force) in order to calculate the expected ultimate tensile force (radial force) of both types of tendons. To explore what tensile forces are to be expected, several studies reporting the tensile forces of tendons were found. Since there is no standardized tissue testing method, the different results of mechanical properties can be noticed [28,29,30,31,32,33]. To fulfil the gripping task, it was decided to design the cylinder with a diameter of 50 mm, which allows the maximum axial force of 1900 N to be achieved. However, gripping in advance has been solved with the new tendon clamping technique presented in [21] using bolt preloads. With this technique, the actuator was used just to hold the clamps together with the tendon within the jaws with 0.3 MPa of pressure. The actuator stroke is 20 mm and it was determined according to the proposed clamping technique and other design elements.

## 6. Conclusions

This work is novel as it presents for the first time the possibilities of using 3D printing FDM technology in the fabrication of a double-acting linear pneumatic actuator. Innovatively, a number of the requirements were met, such as submerging and implementation in the existing workspace of the machine without interrupting the function and functionality of the tensile testing machine. Furthermore, the actuator is presented as a non-commercial device as an integral part of the equipment for tissue testing. This manuscript, together with [26], brings a new refreshment in the relationship between mechanical engineering and medicine. The main contributions and novelties are:

- Three-dimensionally printed actuator for tensile testing of human tendons;
- A fully functional 3D printed actuator, submerged in the test liquid during tendon testing;
- Using the suggested parameters for the actuator FDM process on the hobby-grade 3D printers;
- Three-dimensional printing has been used for the first time to manufacture conventional pneumatic actuator, such as the piston-cylinder assembly, that can operate at standard pneumatic supply pressures.

In the future, different materials for 3D printing the parts of the actuator will be investigated, focusing on the cylinder, piston and end cap. Furthermore, different 3D printing techniques, as well as the types of 3D printers, will be considered. Overall, the robust design needs to be reconsidered and we will try to avoid any reinforcements of the 3D printed structures.

Finally, it was shown that it is possible to design and fabricate a 3D printed actuator that can be used for medical research. This study could open new frontiers in the field of tissue testing and the development of specific devices for such a purpose.

## Figures and Tables

**Table 1:** The main parameters of the actuator FDM process.

Parameters	Value
Layer Height	0.01 mm
Shell Thickness	0.4 mm
Overlap Percentage	40%
Infill Density	100%
Printing Temperature	200 °C
Infill Speed	20 mm/s
Wall Speed	10 mm/s
Build Plate Temperature	70 °C

## Data Availability

Not applicable.
